# Real-time intelligent pattern recognition algorithm for surface EMG signals

**DOI:** 10.1186/1475-925X-6-45

**Published:** 2007-12-03

**Authors:** Mahdi Khezri, Mehran Jahed

**Affiliations:** 1 Sharif University of Technology, Electrical Engineering Department, Biomedical Engineering Group, Tehran, Iran

## Abstract

**Background:**

Electromyography (EMG) is the study of muscle function through the inquiry of electrical signals that the muscles emanate. EMG signals collected from the surface of the skin (Surface Electromyogram: sEMG) can be used in different applications such as recognizing musculoskeletal neural based patterns intercepted for hand prosthesis movements. Current systems designed for controlling the prosthetic hands either have limited functions or can only be used to perform simple movements or use excessive amount of electrodes in order to achieve acceptable results. In an attempt to overcome these problems we have proposed an intelligent system to recognize hand movements and have provided a user assessment routine to evaluate the correctness of executed movements.

**Methods:**

We propose to use an intelligent approach based on adaptive neuro-fuzzy inference system (ANFIS) integrated with a real-time learning scheme to identify hand motion commands. For this purpose and to consider the effect of user evaluation on recognizing hand movements, vision feedback is applied to increase the capability of our system. By using this scheme the user may assess the correctness of the performed hand movement. In this work a hybrid method for training fuzzy system, consisting of back-propagation (BP) and least mean square (LMS) is utilized. Also in order to optimize the number of fuzzy rules, a subtractive clustering algorithm has been developed. To design an effective system, we consider a conventional scheme of EMG pattern recognition system. To design this system we propose to use two different sets of EMG features, namely time domain (TD) and time-frequency representation (TFR). Also in order to decrease the undesirable effects of the dimension of these feature sets, principle component analysis (PCA) is utilized.

**Results:**

In this study, the myoelectric signals considered for classification consists of six unique hand movements. Features chosen for EMG signal are time and time-frequency domain. In this work we demonstrate the capability of an EMG pattern recognition system using ANFIS as classifier with a real-time learning method. Our results reveal that the utilized real-time ANFIS approach along with the user evaluation provides a 96.7% average accuracy. This rate is superior to the previously reported result utilizing artificial neural networks (ANN) real-time method [1].

**Conclusion:**

This study shows that ANFIS real-time learning method coupled with mixed time and time-frequency features as EMG features can provide acceptable results for designing sEMG pattern recognition system suitable for hand prosthesis control.

## Background

The EMG signal provides us with information about the neuromuscular activity from which it originates. This has been fundamental to its use in clinical diagnosis, and as a source for control of assistive devices. It has been proposed that the EMG signals from upper limb musculature can be used to identify motion commands for the control of an externally powered prosthesis hand. Electromyogram signal is a simple way for obtaining necessary information on what the disabled user would like to do with his/her hands. It is possible to control a prosthetic device only with pair of surface mounted differential electrodes placed on residual limbs. [2]

EMG is a complicated signal influenced by various factors such as physiological and anatomical properties and characteristics of instrumentation. It differs from one person to another. In earlier studies, the recognition system learned the characteristics of EMG signal in an offline manner. The offline approach was incapable of adjusting its inner states to correspond to real-time operator's variations of hand movements [3-5]. To decrease these effects on EMG pattern recognition system and to eliminate its dependence on individual subjects, real-time training methods were introduced [1].

Current prosthesis hands, such as Otto Bock [6] commercial hand are unable to provide human-like grasping functionality or deliver motor sensory feedback to the users. Moreover they require a great deal of training and adjusting processes. Also during recent years, several robotic and anthropomorphic hands have been developed such as Utah/MIT hand [7], the Stanford/JPL hand [8], the DLR hand [9], and the Robonaut hand [10]. All of these hands have a high number of degree of freedom and flexibility with non-distinctive hand movements. Moreover they can not truly operate as prosthesis hands due to their heavy and bulky structure and crude gripping functionality. A recent remedy to overcome these shortcomings has been to reduce the degree of freedom [11,12].

Accurate feature extraction from EMG signals is the main kernel of classification system in both real-time and offline systems and is essential to the motion command identification. The non-stationary nature of sEMG signal makes it difficult to precisely extract feature parameters with such block processing stationary models like autoregressive (AR) model [13]. Previous works have shown that time-frequency features present better results in EMG pattern recognition applications [5]. This is due to the effect of combining time domain and frequency analyses which yields a potentially more revealing picture of the temporal localization of a signal's spectral characteristics. However it is very difficult to utilize only one feature set to adequately reflect the unique feature of the measured sEMG signals to a motion command. Therefore in order to increase the recognition rate of this system, we propose to use the feature set based on combined features. Once the feature set is constructed, it is fed to a classifier to discriminate between our proposed six hand motions.

**Figure 1:**
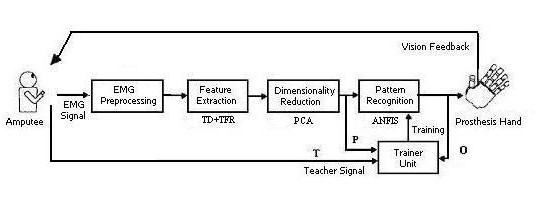
Real-Time Scheme for hand prosthesis control.

The real-time scheme of EMG pattern recognition system used in this work is shown in Figure 1. The five major components are, EMG pre-processing and conditioning, feature extraction, Dimensionality-reduction, classifier (pattern recognizer) and trainer units. The goal of pre-processing step is to prepare and amplify the signal for the subsequent steps and to reduce noise artifacts. This work consists of a feature extraction step followed by a dimensionality reduction technique, namely PCA to simplify the task of the classifier. The role of dimensionality reduction is to retain information that is important for class discrimination and discard irrelevant information. Next a real-time intelligent classifier approach, namely ANFIS is introduced. Finally a novel trainer unit is utilized to relate actual EMG patterns with generated control commands and furthermore to adapt to the operator's characteristics.

### sEMG pattern recognition using adaptive neuro- fuzzy inference system

Fuzzy inference system was developed in 1965 by professor lotfizadeh [14,15]. Fuzzy logic systems can emulate human decision-making more closely than many other classifiers, because of the possibility of introducing the knowledge of an expert system in the fuzzy rules of the form IF-THEN [16-19]. The non-stationary nature of sEMG signal like other biological signal makes the task of classification more difficult. But the characteristics of fuzzy inference system make it a viable tool for pattern recognition applications [20]. The fuzzy system, initially fuzzifies inputs to values at interval [0, 1] using a set of membership functions (MF). Next it is inferred by fuzzy logic through rules in the form of IF-THEN. The basic part of fuzzy system is the fuzzy inference engine that can be used for creating fuzzy rules. The example of fuzzy rules is:

Rj:if x1 is MF1i and/or x2 is MF2i and/or...xj is MFji then zi is MFoi
 MathType@MTEF@5@5@+=feaafiart1ev1aaatCvAUfKttLearuWrP9MDH5MBPbIqV92AaeXatLxBI9gBaebbnrfifHhDYfgasaacPC6xNi=xI8qiVKYPFjYdHaVhbbf9v8qqaqFr0xc9vqFj0dXdbba91qpepeI8k8fiI+fsY=rqGqVepae9pg0db9vqaiVgFr0xfr=xfr=xc9adbaqaaeGacaGaaiaabeqaaeqabiWaaaGcbaGaemOuai1aaWbaaSqabeaacqWGQbGAaaGccqGG6aGocqqGPbqAcqqGMbGzcqqGGaaicqWG4baEdaWgaaWcbaGaeGymaedabeaakiabbccaGiabbMgaPjabbohaZjabbccaGiabd2eanjabdAeagnaaDaaaleaacqaIXaqmaeaacqWGPbqAaaGccqqGGaaicqqGHbqycqqGUbGBcqqGKbazcqqGVaWlcqqGVbWBcqqGYbGCcqqGGaaicqWG4baEdaWgaaWcbaGaeGOmaidabeaakiabbccaGiabbMgaPjabbohaZjabbccaGiabd2eanjabdAeagnaaDaaaleaacqaIYaGmaeaacqWGPbqAaaGccqqGGaaicqqGHbqycqqGUbGBcqqGKbazcqqGVaWlcqqGVbWBcqqGYbGCcqGGUaGlcqGGUaGlcqGGUaGlcqWG4baEdaWgaaWcbaGaemOAaOgabeaakiabbccaGiabbMgaPjabbohaZjabbccaGiabd2eanjabdAeagnaaDaaaleaacqWGQbGAaeaacqWGPbqAaaGccqqGGaaicqqG0baDcqqGObaAcqqGLbqzcqqGUbGBcqqGGaaicqWG6bGEdaahaaWcbeqaaiabdMgaPbaakiabbccaGiabbMgaPjabbohaZjabbccaGiabd2eanjabdAeagjabd+gaVnaaCaaaleqabaGaemyAaKgaaaaa@7F29@

where *R*^*j *^(i = 1,2, .., l) denotes the *i*^*th *^fuzzy rules, *x*_*j *_(j = 1,2, .., n) is the *j*^*th *^input and *z*^*i *^is the output of *j*^*th *^fuzzy rule, and MFji,MFoi
 MathType@MTEF@5@5@+=feaafiart1ev1aqatCvAUfKttLearuWrP9MDH5MBPbIqV92AaeXatLxBI9gBaebbnrfifHhDYfgasaacPC6xNi=xH8viVGI8Gi=hEeeu0xXdbba9frFj0xb9qqpG0dXdb9aspeI8k8fiI+fsY=rqGqVepae9pg0db9vqaiVgFr0xfr=xfr=xc9adbaqaaeGacaGaaiaabeqaaeqabiWaaaGcbaGaemyta0KaemOray0aa0baaSqaaiabdQgaQbqaaiabdMgaPbaakiabcYcaSiabd2eanjabdAeagjabd+gaVnaaCaaaleqabaGaemyAaKgaaaaa@3702@ are fuzzy membership function of antecedents and consequents for *i*^*th *^rules.

**Figure 2:**
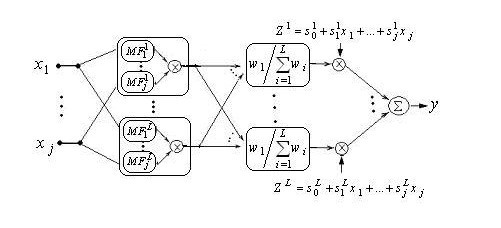
Network representing ANFIS structure. (MFs are bell membership functions).

In this work we applied a neuro-fuzzy scheme to recognize sEMG patterns. Neuro-fuzzy computing enables us to build a more robust intelligent based decision making systems by combining the advantage of artificial neural network with the fuzzy modeling of Imprecise and qualitative knowledge. Figure 2 depicts the ANFIS structure with n inputs and one output. The output of this system can be described by the following function:

y=∑i=1L{(∏j=1nMFji(xj)).(zi)∑i=1L(∏j=1nMFji(xj))}
 MathType@MTEF@5@5@+=feaafiart1ev1aaatCvAUfKttLearuWrP9MDH5MBPbIqV92AaeXatLxBI9gBaebbnrfifHhDYfgasaacPC6xNi=xI8qiVKYPFjYdHaVhbbf9v8qqaqFr0xc9vqFj0dXdbba91qpepeI8k8fiI+fsY=rqGqVepae9pg0db9vqaiVgFr0xfr=xfr=xc9adbaqaaeGacaGaaiaabeqaaeqabiWaaaGcbaGaemyEaKNaeyypa0ZaaabCaeaadaGadaqcfayaamaalaaabaWaaeWaaeaadaqeWbqaaiabd2eanjabdAeagnaaDaaabaGaemOAaOgabaGaemyAaKgaaiabcIcaOiabdIha4naaBaaabaGaemOAaOgabeaacqGGPaqkaeaacqWGQbGAcqGH9aqpcqaIXaqmaeaacqWGUbGBaiabg+GivdaacaGLOaGaayzkaaGaeiOla4YaaeWaaeaacqWG6bGEdaahaaqabeaacqWGPbqAaaaacaGLOaGaayzkaaaabaWaaabCaeaadaqadaqaamaarahabaGaemyta0KaemOray0aa0baaeaacqWGQbGAaeaacqWGPbqAaaGaeiikaGIaemiEaG3aaSbaaeaacqWGQbGAaeqaaiabcMcaPaqaaiabdQgaQjabg2da9iabigdaXaqaaiabd6gaUbGaey4dIunaaiaawIcacaGLPaaaaeaacqWGPbqAcqGH9aqpcqaIXaqmaeaacqWGmbataiabggHiLdaaaaGccaGL7bGaayzFaaaaleaacqWGPbqAcqGH9aqpcqaIXaqmaeaacqWGmbata0GaeyyeIuoaaaa@685C@

where MF is the membership function. In this work we chose the generalized bell function as membership function. This function depends on three parameters namely a, b, c as given by:

MF(x)=11+|x−ca|2b
 MathType@MTEF@5@5@+=feaafiart1ev1aaatCvAUfKttLearuWrP9MDH5MBPbIqV92AaeXatLxBI9gBaebbnrfifHhDYfgasaacPC6xNi=xI8qiVKYPFjYdHaVhbbf9v8qqaqFr0xc9vqFj0dXdbba91qpepeI8k8fiI+fsY=rqGqVepae9pg0db9vqaiVgFr0xfr=xfr=xc9adbaqaaeGacaGaaiaabeqaaeqabiWaaaGcbaGaemyta0KaemOrayKaeiikaGIaemiEaGNaeiykaKIaeyypa0tcfa4aaSaaaeaacqaIXaqmaeaacqaIXaqmcqGHRaWkdaabdaqaamaalaaabaGaemiEaGNaeyOeI0Iaem4yamgabaGaemyyaegaaaGaay5bSlaawIa7amaaCaaabeqaaiabikdaYiabdkgaIbaaaaaaaa@407D@

The basic problem of fuzzy system is, adjusting membership function parameters, output of each fuzzy rule and estimating number of rules that should be minimum and precise enough. Adaptive neuro-fuzzy inference system adapts the parameters of Sugeno type inference system using the neural networks [21]. For Sugeno type systems, output is a crisp number computed by multiplying each input by a constant and then adding up the results. The resultant output is in the form of z1=s01+s11x1+...+sj1xj
 MathType@MTEF@5@5@+=feaafiart1ev1aaatCvAUfKttLearuWrP9MDH5MBPbIqV92AaeXatLxBI9gBaebbnrfifHhDYfgasaacPC6xNi=xH8viVGI8Gi=hEeeu0xXdbba9frFj0xb9qqpG0dXdb9aspeI8k8fiI+fsY=rqGqVepae9pg0db9vqaiVgFr0xfr=xfr=xc9adbaqaaeGacaGaaiaabeqaaeqabiWaaaGcbaGaemOEaO3aaWbaaSqabeaacqaIXaqmaaGccqGH9aqpcqWGZbWCdaqhaaWcbaGaeGimaadabaGaeGymaedaaOGaey4kaSIaem4Cam3aa0baaSqaaiabigdaXaqaaiabigdaXaaakiabdIha4naaBaaaleaacqaIXaqmaeqaaOGaey4kaSIaeiOla4IaeiOla4IaeiOla4Iaey4kaSIaem4Cam3aa0baaSqaaiabdQgaQbqaaiabigdaXaaakiabdIha4naaBaaaleaacqWGQbGAaeqaaaaa@456D@.

For training fuzzy system, ANFIS employs BP scheme for the parameters associated with the input membership functions, and LMS estimation for the parameters associated with the output membership functions.

In order to optimize the fuzzy system and increase its ability for sEMG pattern recognition problem, subtractive clustering was employed to optimize fuzzy rules specification. This method partitions the data into groups called clusters, and generates a Fuzzy Inference System (FIS) with the minimum number of rules required to distinguish the fuzzy qualities associated with each of the clusters. In the next section we introduce these methods and describe their application in implementing neuro-fuzzy system.

### Hybrid method (BP and LMS)

The ANFIS structure can be used for training fuzzy inference system. One of the most useful algorithms that can be used for this purpose is back-propagation. BP adjusts membership function parameters. For neuro-fuzzy system usually the bell function is applied as membership function. In this function a, b and c are considered variables and must be adjusted. The BP algorithm may be used to train these parameters. Suppose that we are given an input-output pair (*x*, *y*), *x *= [*x*_1_, *x*_2_, ... *x*_*n*_], our goal is, to minimize the cost function (where *y*_*des *_is desired output):

e=12[ydes−y]2
 MathType@MTEF@5@5@+=feaafiart1ev1aaatCvAUfKttLearuWrP9MDH5MBPbIqV92AaeXatLxBI9gBaebbnrfifHhDYfgasaacPC6xNi=xI8qiVKYPFjYdHaVhbbf9v8qqaqFr0xc9vqFj0dXdbba91qpepeI8k8fiI+fsY=rqGqVepae9pg0db9vqaiVgFr0xfr=xfr=xc9adbaqaaeGacaGaaiaabeqaaeqabiWaaaGcbaGaemyzauMaeyypa0tcfa4aaSaaaeaacqaIXaqmaeaacqaIYaGmaaGcdaWadaqaaiabdMha5naaBaaaleaacqWGKbazcqWGLbqzcqWGZbWCaeqaaOGaeyOeI0IaemyEaKhacaGLBbGaayzxaaWaaWbaaSqabeaacqaIYaGmaaaaaa@3C41@

The output of each rule *z^i^* is defined by:

zi(t+1)=zi(t)−kz∂e∂zi
 MathType@MTEF@5@5@+=feaafiart1ev1aaatCvAUfKttLearuWrP9MDH5MBPbIqV92AaeXatLxBI9gBaebbnrfifHhDYfgasaacPC6xNi=xI8qiVKYPFjYdHaVhbbf9v8qqaqFr0xc9vqFj0dXdbba91qpepeI8k8fiI+fsY=rqGqVepae9pg0db9vqaiVgFr0xfr=xfr=xc9adbaqaaeGacaGaaiaabeqaaeqabiWaaaGcbaGaemOEaO3aaWbaaSqabeaacqWGPbqAaaGccqGGOaakcqWG0baDcqGHRaWkcqaIXaqmcqGGPaqkcqGH9aqpcqWG6bGEdaahaaWcbeqaaiabdMgaPbaakiabcIcaOiabdsha0jabcMcaPiabgkHiTiabdUgaRnaaBaaaleaacqWG6bGEaeqaaKqbaoaalaaabaGaeyOaIyRaemyzaugabaGaeyOaIyRaemOEaO3aaWbaaeqabaGaemyAaKgaaaaaaaa@4709@

where *k*_*z *_is a step size. To adjust a, b, and c parameters, we start with Sugeno's system. Here, we can specify the output as follows:

wi=∏j=1nMFj,y=∑i=1Lwizi∑i=1Lwi
 MathType@MTEF@5@5@+=feaafiart1ev1aaatCvAUfKttLearuWrP9MDH5MBPbIqV92AaeXatLxBI9gBaebbnrfifHhDYfgasaacPC6xNi=xI8qiVKYPFjYdHaVhbbf9v8qqaqFr0xc9vqFj0dXdbba91qpepeI8k8fiI+fsY=rqGqVepae9pg0db9vqaiVgFr0xfr=xfr=xc9adbaqaaeGacaGaaiaabeqaaeqabiWaaaGcbaqbaeqabeGaaaqaaiabdEha3naaCaaaleqabaGaemyAaKgaaOGaeyypa0ZaaebCaeaacqWGnbqtcqWGgbGrdaWgaaWcbaGaemOAaOgabeaaaeaacqWGQbGAcqGH9aqpcqaIXaqmaeaacqWGUbGBa0Gaey4dIunakiabcYcaSaqaaiabdMha5jabg2da9KqbaoaalaaabaWaaabCaeaacqWG3bWDdaahaaqabeaacqWGPbqAaaGaemOEaO3aaWbaaeqabaGaemyAaKgaaaqaaiabdMgaPjabg2da9iabigdaXaqaaiabdYeambGaeyyeIuoaaeaadaaeWbqaaiabdEha3naaCaaabeqaaiabdMgaPbaaaeaacqWGPbqAcqGH9aqpcqaIXaqmaeaacqWGmbataiabggHiLdaaaaaaaaa@54FF@

Derivative of equation (4) based on equation (6) is given by:

∂e∂zi=∂e∂y⋅∂y∂zi,∂y∂zi=wi∑i=1Lwi and ∂e∂y=(ydes−y)
 MathType@MTEF@5@5@+=feaafiart1ev1aaatCvAUfKttLearuWrP9MDH5MBPbIqV92AaeXatLxBI9gBaebbnrfifHhDYfgasaacPC6xNi=xI8qiVKYPFjYdHaVhbbf9v8qqaqFr0xc9vqFj0dXdbba91qpepeI8k8fiI+fsY=rqGqVepae9pg0db9vqaiVgFr0xfr=xfr=xc9adbaqaaeGacaGaaiaabeqaaeqabiWaaaGcbaqcfa4aaSaaaeaacqGHciITcqWGLbqzaeaacqGHciITcqWG6bGEdaahaaqabeaacqWGPbqAaaaaaOGaeyypa0tcfa4aaSaaaeaacqGHciITcqWGLbqzaeaacqGHciITcqWG5bqEaaGaeyyXIC9aaSaaaeaacqGHciITcqWG5bqEaeaacqGHciITcqWG6bGEdaahaaqabeaacqWGPbqAaaaaaiabcYcaSmaalaaabaGaeyOaIyRaemyEaKhabaGaeyOaIyRaemOEaO3aaWbaaeqabaGaemyAaKgaaaaakiabg2da9KqbaoaalaaabaGaem4DaC3aaWbaaeqabaGaemyAaKgaaaqaamaaqahabaGaem4DaC3aaWbaaeqabaGaemyAaKgaaaqaaiabdMgaPjabg2da9iabigdaXaqaaiabdYeambGaeyyeIuoaaaGaeeiiaaIccqqGHbqycqqGUbGBcqqGKbazcqqGGaaijuaGdaWcaaqaaiabgkGi2kabdwgaLbqaaiabgkGi2kabdMha5baakiabg2da9iabcIcaOiabdMha5naaBaaaleaacqWGKbazcqWGLbqzcqWGZbWCaeqaaOGaeyOeI0IaemyEaKNaeiykaKcaaa@71D7@

Then for output of each rule we can define the following equations:

zi(t+1)=zi(t)−kz(wi∑i=1Lwi)(ydes−y)
 MathType@MTEF@5@5@+=feaafiart1ev1aaatCvAUfKttLearuWrP9MDH5MBPbIqV92AaeXatLxBI9gBaebbnrfifHhDYfgasaacPC6xNi=xI8qiVKYPFjYdHaVhbbf9v8qqaqFr0xc9vqFj0dXdbba91qpepeI8k8fiI+fsY=rqGqVepae9pg0db9vqaiVgFr0xfr=xfr=xc9adbaqaaeGacaGaaiaabeqaaeqabiWaaaGcbaGaemOEaO3aaWbaaSqabeaacqWGPbqAaaGccqGGOaakcqWG0baDcqGHRaWkcqaIXaqmcqGGPaqkcqGH9aqpcqWG6bGEdaahaaWcbeqaaiabdMgaPbaakiabcIcaOiabdsha0jabcMcaPiabgkHiTiabdUgaRnaaBaaaleaacqWG6bGEaeqaaOGaeiikaGscfa4aaSaaaeaacqWG3bWDdaahaaqabeaacqWGPbqAaaaabaWaaabCaeaacqWG3bWDdaahaaqabeaacqWGPbqAaaaabaGaemyAaKMaeyypa0JaeGymaedabaGaemitaWeacqGHris5aaaakiabcMcaPiabcIcaOiabdMha5naaBaaaleaacqWGKbazcqWGLbqzcqWGZbWCaeqaaOGaeyOeI0IaemyEaKNaeiykaKcaaa@5818@

Similarly, for *j*^*th *^membership function of *i*^*th *^fuzzy rule the parameters are calculated as:

aji(t+1)=aji(t)−ka∂e∂aji
 MathType@MTEF@5@5@+=feaafiart1ev1aaatCvAUfKttLearuWrP9MDH5MBPbIqV92AaeXatLxBI9gBaebbnrfifHhDYfgasaacPC6xNi=xI8qiVKYPFjYdHaVhbbf9v8qqaqFr0xc9vqFj0dXdbba91qpepeI8k8fiI+fsY=rqGqVepae9pg0db9vqaiVgFr0xfr=xfr=xc9adbaqaaeGacaGaaiaabeqaaeqabiWaaaGcbaGaemyyae2aa0baaSqaaiabdQgaQbqaaiabdMgaPbaakiabcIcaOiabdsha0jabgUcaRiabigdaXiabcMcaPiabg2da9iabdggaHnaaDaaaleaacqWGQbGAaeaacqWGPbqAaaGccqGGOaakcqWG0baDcqGGPaqkcqGHsislcqWGRbWAdaWgaaWcbaGaemyyaegabeaajuaGdaWcaaqaaiabgkGi2kabdwgaLbqaaiabgkGi2kabdggaHnaaDaaabaGaemOAaOgabaGaemyAaKgaaaaaaaa@4A58@

bji(t+1)=bji(t)−kb∂e∂bji
 MathType@MTEF@5@5@+=feaafiart1ev1aaatCvAUfKttLearuWrP9MDH5MBPbIqV92AaeXatLxBI9gBaebbnrfifHhDYfgasaacPC6xNi=xI8qiVKYPFjYdHaVhbbf9v8qqaqFr0xc9vqFj0dXdbba91qpepeI8k8fiI+fsY=rqGqVepae9pg0db9vqaiVgFr0xfr=xfr=xc9adbaqaaeGacaGaaiaabeqaaeqabiWaaaGcbaGaemOyai2aa0baaSqaaiabdQgaQbqaaiabdMgaPbaakiabcIcaOiabdsha0jabgUcaRiabigdaXiabcMcaPiabg2da9iabdkgaInaaDaaaleaacqWGQbGAaeaacqWGPbqAaaGccqGGOaakcqWG0baDcqGGPaqkcqGHsislcqWGRbWAdaWgaaWcbaGaemOyaigabeaajuaGdaWcaaqaaiabgkGi2kabdwgaLbqaaiabgkGi2kabdkgaInaaDaaabaGaemOAaOgabaGaemyAaKgaaaaaaaa@4A60@

cji(t+1)=cji(t)−kb∂e∂cji
 MathType@MTEF@5@5@+=feaafiart1ev1aaatCvAUfKttLearuWrP9MDH5MBPbIqV92AaeXatLxBI9gBaebbnrfifHhDYfgasaacPC6xNi=xI8qiVKYPFjYdHaVhbbf9v8qqaqFr0xc9vqFj0dXdbba91qpepeI8k8fiI+fsY=rqGqVepae9pg0db9vqaiVgFr0xfr=xfr=xc9adbaqaaeGacaGaaiaabeqaaeqabiWaaaGcbaGaem4yam2aa0baaSqaaiabdQgaQbqaaiabdMgaPbaakiabcIcaOiabdsha0jabgUcaRiabigdaXiabcMcaPiabg2da9iabdogaJnaaDaaaleaacqWGQbGAaeaacqWGPbqAaaGccqGGOaakcqWG0baDcqGGPaqkcqGHsislcqWGRbWAdaWgaaWcbaGaemOyaigabeaajuaGdaWcaaqaaiabgkGi2kabdwgaLbqaaiabgkGi2kabdogaJnaaDaaabaGaemOAaOgabaGaemyAaKgaaaaaaaa@4A66@

In order to specify the number of rules in fuzzy system we utilize subtractive clustering approach. This method is introduced bellow.

### Subtractive clustering

Subtractive clustering is based on a measure of the density of data points in the feature space. The idea behind this approach is to find regions in the feature space with high densities of data points. The point with the highest number of neighbours is selected as the center for a cluster. The data points within a prespecified fuzzy radius are then removed (subtracted), and the algorithm looks for a new point with the highest number of neighbours. This continues until all data points are examined. Consider a collection of K data points specified by m-dimensional vectors *u*_*k*_, k = 1, 2..., K. Since each data point is a candidate for a cluster center, a density measure at data point *u*_*k *_is defined as:

Dk=∑j=1Kexp⁡(−‖uk−uj‖(ra/2)2)
 MathType@MTEF@5@5@+=feaafiart1ev1aaatCvAUfKttLearuWrP9MDH5MBPbIqV92AaeXatLxBI9gBaebbnrfifHhDYfgasaacPC6xNi=xI8qiVKYPFjYdHaVhbbf9v8qqaqFr0xc9vqFj0dXdbba91qpepeI8k8fiI+fsY=rqGqVepae9pg0db9vqaiVgFr0xfr=xfr=xc9adbaqaaeGacaGaaiaabeqaaeqabiWaaaGcbaGaemiraq0aaSbaaSqaaiabdUgaRbqabaGccqGH9aqpdaaeWbqaaiGbcwgaLjabcIha4jabcchaWnaabmaabaGaeyOeI0scfa4aaSaaaeaadaqbdaqaaiabdwha1naaBaaabaGaem4AaSgabeaacqGHsislcqWG1bqDdaWgaaqaaiabdQgaQbqabaaacaGLjWUaayPcSdaabaGaeiikaGYaaSGbaeaacqWGYbGCdaWgaaqaaiabdggaHbqabaaabaGaeGOmaiJaeiykaKYaaWbaaeqabaGaeGOmaidaaaaaaaaakiaawIcacaGLPaaaaSqaaiabdQgaQjabg2da9iabigdaXaqaaiabdUealbqdcqGHris5aaaa@4E72@

where *r*_*a *_is a positive constant. Hence, a data point will have a high density value if it has many neighbouring data points. Only the fuzzy neighborhood within the radius of *r*_*a *_contributes to the density measure. After calculating the density measure for each data point, the point with the highest density is selected as the first cluster center. Let *u*_*c*1 _be the point selected and *D*_*c*1 _its density measure. Next, the density measure for each data point *u*_*k *_is revised by the formula:

Dk'=Dk−Dc1exp⁡(−‖uk−uc1‖(rb/2)2)
 MathType@MTEF@5@5@+=feaafiart1ev1aaatCvAUfKttLearuWrP9MDH5MBPbIqV92AaeXatLxBI9gBaebbnrfifHhDYfgasaacPC6xNi=xI8qiVKYPFjYdHaVhbbf9v8qqaqFr0xc9vqFj0dXdbba91qpepeI8k8fiI+fsY=rqGqVepae9pg0db9vqaiVgFr0xfr=xfr=xc9adbaqaaeGacaGaaiaabeqaaeqabiWaaaGcbaGaemiraq0aa0baaSqaaiabdUgaRbqaaiabcEcaNaaakiabg2da9iabdseaenaaBaaaleaacqWGRbWAaeqaaOGaeyOeI0Iaemiraq0aaSbaaSqaaiabdogaJjabigdaXaqabaGccyGGLbqzcqGG4baEcqGGWbaCdaqadaqaaiabgkHiTKqbaoaalaaabaWaauWaaeaacqWG1bqDdaWgaaqaaiabdUgaRbqabaGaeyOeI0IaemyDau3aaSbaaeaacqWGJbWydaWgaaqaaiabigdaXaqabaaabeaaaiaawMa7caGLkWoaaeaacqGGOaakdaWcgaqaaiabdkhaYnaaBaaabaGaemOyaigabeaaaeaacqaIYaGmaaGaeiykaKYaaWbaaeqabaGaeGOmaidaaaaaaOGaayjkaiaawMcaaaaa@50B3@

where *r*_*b *_is a positive constant. Therefore, the data points near the first cluster center *u*_*c*1 _will have significantly reduced density measures, thereby making the points unlikely to be selected as the next cluster center. The constant *r*_*b *_defines a neighborhood to be reduced in density measure and it is normally larger than *r*_*a *_to prevent closely spaced cluster centers, where typically *r*_*b *_= 1.5 × *r*_*a*_. After the density measure for each point is revised, the next cluster center *u*_*c*2 _is selected and all the density measures are revised again. The process is repeated until a sufficient number of cluster centers are generated. When applying subtractive clustering to a set of input-output data, each of the cluster centers represents a rule. To generate rules, the cluster centers are used as the centers for the premise sets in a singleton type of rule base (or the radial basis functions in a radial basis function neural network).

## Methods

### EMG acquisition and pre-processing

The EMG signal is the electrical manifestation of the neuromuscular activation associated with a contracting muscle. A good acquisition of the sEMG signal is a prerequisite for good signal processing. Since hand motions result from contraction of the muscles in the forearm section, we used surface electrodes for measuring sEMG signal from the extensor digitorum, the extensor carpi radialis, the palmaris longus and the flexor carpi ulnaris. In this work we use two channels of differential surface electrodes for collecting sEMG signal.

This Signal is easily affected by undesired signal that come from different sources such as 50/60 Hz electromagnetic interference from power lines. In addition, for surface electrode instrumentation, complicating issues may arise due to its coupling with skin. Concerns such as impedance of the skin, its superficial oil content and the density of its dead cell layer are to name a few. We placed differential electrodes on the forearm under the elbow and placed reference electrode on the wrist. After the acquisition, sEMG signal was filtered using a band-pass filter consisting of a high-pass filter with 500 Hz cut off frequency to reduce motion artifacts and a low pass filter of 20 Hz cut-off frequency to reduce noise. The signal was next amplified with a high common mode rejection ratio (CMRR) amplifier [22]. Also we exerted a notch filter at 50 Hz to eliminate power line noise. Finally the signal was sampled at 1 KHz and transferred to an IBM based personal computer (PC) for further analysis.

**Figure 3:**
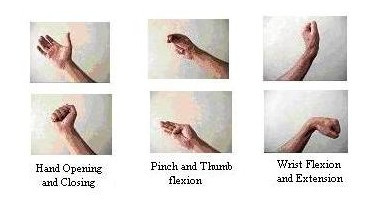
The six classes of movements used in this work.

A roster of four healthy subjects participated for collecting sEMG signals. Six hand movements were considered and sEMG signal for each was extracted. These movements depicted in Figure 3, were Hand opening and closing, pinch, thumb flexion, wrist flexion and extension. For each class of movement, 100 signals were collected. We divided the acquired signals into two categories. First category was utilized as a training set data and the second was employed as a test set, in the manner that each of them included 50 signals. To increase the ability of our system to recognize hand movements, we utilized several signals in each class of movement as a validation set and therefore the test set actually incorporated both the test and validation sets. In this work training and test data sets were formed by 300 samples, or 50 samples for each class. Table 1 presents the class distribution of the samples in the training and validation data sets.

**Table 1:** Class distribution of the sample in training and test set data

**Class (Motion's type)**	**Training set**	**Test set**	**Total**
1-Opening	50	50	100
2-Closing	50	50	100
3-Wrist flexion	50	50	100
4-Wrist extension	50	50	100
5-Pinch	50	50	100
6-Thumb flexion	50	50	100
Total	300	300	600

### Features selection

Time domain features extracts time structures in the EMG signal. The EMG signal has a number of irregular structures in the temporal waveform due to its deterministic components but it also exhibits a great deal of intraclass variability due to existence of random component. In our analysis various features of the signal were considered. Sampled waveform for sEMG pattern classification will result in the undesirable loss of temporal structure of the signal. Moreover, in this case, due to high variability of features and high dimension of feature space, a further poor classification performance will occur.

To overcome the loss of temporal information of the signal and poor classification performance, we segmented sEMG signal and extracted desirable features from each segment. In this work, we used a time domain window of 500 (ms) for collecting sEMG signal. For classification problem, appropriate length of sEMG signal should be considered. Hudgins experimented with different segment lengths in an attempt to reduce classification error. He decided on a scheme of five 40 (ms) segment plus an extra segment [3].

**Figure 4:**
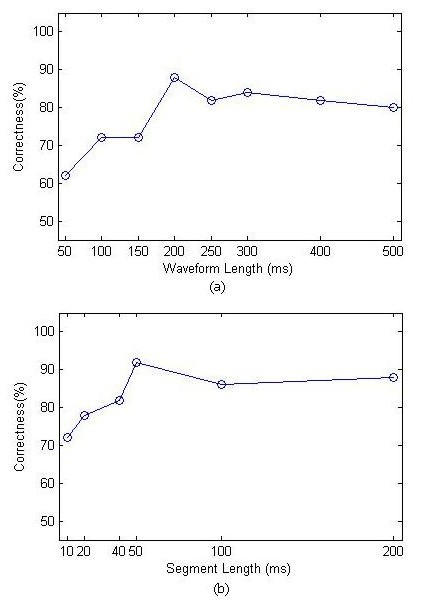
Selection of sEMG waveform length and segment number to construct time domain feature set.

In this work, 200 (ms) segmented signal was found to be most appropriate for the classification problem. We decided on four 50 ms sub-segment for better accuracy. Figure 4 shows the selected wavelength signal and segment length for the task of sEMG pattern recognition. EMG features must be calculated over all four segments. In this study we used, mean absolute value (MAV), slope sign changes (SSC) and AR model coefficients as time features of the signal.

As noted earlier, in order to increase the recognition rate of the system, we incorporated the time-frequency features. The major incentive for using these representations as a feature for the application of sEMG pattern recognition is the need for more signal information to better discriminate amongst various hand movements. Discrete wavelet transform (DWT) is a well-known type of time-frequency representation. Wavelet coefficients can be very effective however there is a fundamental drawback namely, lack of shift invariance. If the signal to be analyzed is shifted, the coefficients of wavelet transform vary in a complex manner. This matter presents a significant problem in the task of pattern recognition. To overcome this problem, we can use shift invariant features of DWT such as zero crossing (ZC) and local maxima [23].

For constructing feature set with DWT, we needed to determine the parameters that most effectively influenced it. For this reason we considered candidate parameters and compared their accuracy in the sEMG pattern recognition system. For DWT two parameters were considered, namely choice of mother wavelet and depth of decomposition. The best mother wavelet for sEMG pattern recognition was determined empirically. Basically, the selection of mother wavelet must be based on best correlation with the EMG signal.

The DWT decomposition can be terminated prior to a full decomposition. If the signal has a length of N samples, then the maximum depth of decomposition is J=log⁡2N
 MathType@MTEF@5@5@+=feaafiart1ev1aaatCvAUfKttLearuWrP9MDH5MBPbIqV92AaeXatLxBI9gBaebbnrfifHhDYfgasaacPC6xNi=xH8viVGI8Gi=hEeeu0xXdbba9frFj0xb9qqpG0dXdb9aspeI8k8fiI+fsY=rqGqVepae9pg0db9vqaiVgFr0xfr=xfr=xc9adbaqaaeGacaGaaiaabeqaaeqabiWaaaGcbaGaemOsaOKaeyypa0JagiiBaWMaei4Ba8Maei4zaC2aa0baaSqaaiabikdaYaqaaiabd6eaobaaaaa@3458@. The sEMG signals in this work had 512 samples thus the maximum depth of decomposition was chosen to be nine. For choosing the best mother wavelet, different mother wavelet types such as Haar, Daubechies, Symlet, Coiflet and Biorthogonal were applied and their performance was accordingly evaluated. These results were obtained by initially selecting the mother wavelet type and using 9 level of decomposition for sEMG signal. Englehart et al [5] showed that high level of decomposition provided best performance of EMG pattern recognition system. This was followed by selecting the best depth of sEMG decomposition based on the selected wavelets from the pervious step. In this stage in accordance with Englehart's work, at high level of decomposition, the best accuracy of the system was obtained.

**Figure 5:**
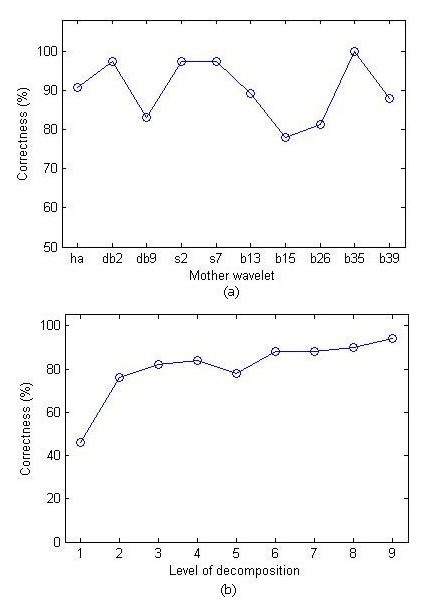
Selection of DWT parameters to construct sEMG feature set.

The results indicate that biorthogonal3.5 as a mother wavelet with 9 level of decomposition, presents the best performance to recognize sEMG patterns. Figure 5 depicts the process of DWT parameters selection.

### Role of trainer unit in real-time pattern recognition

For implementation of a real-time learning scheme in pattern recognition applications, operator's evaluation of system performance is required. In the case of offline methods previously mentioned, there is no need for considering the time as a cost function.

The real-time method must be able to evaluate the accuracy of system in recognizing hand movement that is performed by operator. The trainer unit constructs the training data which contains a teacher reference signal from the operator and reduced features from dimensionality reduction unit. Next, this training data is fed to the pattern recognition unit. When the trainer unit receives the teacher reference signal from the operator, it creates the teaching vector as,

t=(t1,t2,...,tn),ti={1,if i=T0,otherwise
 MathType@MTEF@5@5@+=feaafiart1ev1aaatCvAUfKttLearuWrP9MDH5MBPbIqV92AaeXatLxBI9gBaebbnrfifHhDYfgasaacPC6xNi=xI8qiVKYPFjYdHaVhbbf9v8qqaqFr0xc9vqFj0dXdbba91qpepeI8k8fiI+fsY=rqGqVepae9pg0db9vqaiVgFr0xfr=xfr=xc9adbaqaaeGacaGaaiaabeqaaeqabiWaaaGcbaqbaeqabeGaaaqaaiabdsha0jabg2da9iabcIcaOiabdsha0naaBaaaleaacqaIXaqmaeqaaOGaeiilaWIaemiDaq3aaSbaaSqaaiabikdaYaqabaGccqGGSaalcqGGUaGlcqGGUaGlcqGGUaGlcqGGSaalcqWG0baDdaWgaaWcbaGaemOBa4gabeaakiabcMcaPiabcYcaSaqaaiabdsha0naaBaaaleaacqWGPbqAaeqaaOGaeyypa0ZaaiqabeaafaqabeGacaaabaGaeGymaeJaeiilaWcabaGaemyAaKMaemOzayMaeeiiaaIaemyAaKMaeyypa0JaemivaqfabaGaeGimaaJaeiilaWcabaGaem4Ba8MaemiDaqNaemiAaGMaemyzauMaemOCaiNaem4DaCNaemyAaKMaem4CamNaemyzaugaaaGaay5Eaaaaaaaa@5B1E@

where T is a desired hand movement and {*T *= 1, 2, ..., *n*} for n total hand motions. The trainer unit creates the reduced feature set (p) with teaching data (t) in the form of Γ(*p*,*t*) and sends this teaching vector to pattern recognition unit. The trainer unit updates the state of pattern recognition unit in the interval that demanded control command from EMG signal is generated. This process is continued until the root mean square (RMS) comparison is within the acceptable range and in this case, the threshold was set at 0.1.

RMS=1n∑i=1n(t−O)2≤0.1
 MathType@MTEF@5@5@+=feaafiart1ev1aaatCvAUfKttLearuWrP9MDH5MBPbIqV92AaeXatLxBI9gBaebbnrfifHhDYfgasaacPC6xNi=xI8qiVKYPFjYdHaVhbbf9v8qqaqFr0xc9vqFj0dXdbba91qpepeI8k8fiI+fsY=rqGqVepae9pg0db9vqaiVgFr0xfr=xfr=xc9adbaqaaeGacaGaaiaabeqaaeqabiWaaaGcbaGaemOuaiLaemyta0Kaem4uamLaeyypa0tcfa4aaSaaaeaacqaIXaqmaeaacqWGUbGBaaGcdaaeWbqaamaabmaabaGaemiDaqNaeyOeI0Iaem4ta8eacaGLOaGaayzkaaaaleaacqWGPbqAcqGH9aqpcqaIXaqmaeaacqWGUbGBa0GaeyyeIuoakmaaCaaaleqabaGaeGOmaidaaOGaeyizImQaeGimaaJaeiOla4IaeGymaedaaa@4553@

### Designing the neuro-fuzzy system structure

As noted before, five features of sEMG signal for the application of pattern recognition in this study, namely MAV, SSC, and AR model are applied as time domain features and number of ZC and local maxima of wavelet coefficients are employed as time-frequency features. Therefore our proposed system has five inputs.

**Figure 6:**
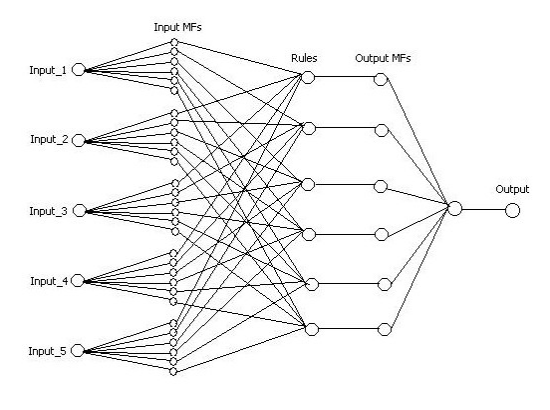
Structure of fuzzy system with five inputs and one output.

In this work a subtractive clustering method is employed to determine the number of fuzzy system rules, while BP and LMS algorithm are utilized for membership function parameters and rule outputs, respectively. For the recognition system that is designed with compound features, six fuzzy rules are employed. As shown before this system is of Sugeno-type of order five and the output for each rule is determined by LMS method. Consider *Z*_*i *_in the form of:

*Z*_*i *_= *α*_*i*1_(*in*1) + *α*_*i*2_(*in*2) + *α*_*i*3_(*in*3) + *α*_*i*4 _(*in*4) + *α*_*i*5 _(*in*5) + *α*_*i*6_

where *in*1 represents the first input and so on. Figure 6 depicts the structure of this fuzzy system. Our proposed system has five inputs, one output and six fuzzy rules. The characteristic of this system is summarized in Table 2. To design this system, we use "prod" as an AND method, "probor" as an OR method, and "weigh average" as a deffuzification operator.

**Table 2:** Characteristic of designed neuro-fuzzy system

**Number of inputs**	**Number of outputs**	**Number of Fuzzy Rules**	**Number of input parameters (MF parameters)**	**Number of output parameters**
5	1	6	90	36

In our study to determine the fuzzy rules, membership functions are represented by linguistic expressions. For this purpose we introduce these expressions from lower to upper range of inputs, as low, middle-low, average-low, average-high, middle-high and high, respectively. The output of each rule is represented as *Z*_*i*_, (for i = 1, 2, 3, 4, 5, 6) and each output is obtained by combining all inputs.

## Results

### Experimental results

**Figure 7:**
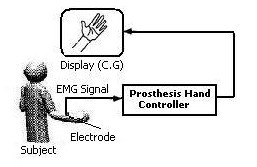
An experimental setup for a real-time EMG pattern recognition system.

To implement the real-time EMG recognition system, we used a PC with a Pentium4 processor. The proposed feedback system was implemented by MATLAB software. Firstly a key on the keyboard was pressed to initiate the teacher signal to the trainer unit. A computer graphic (C.G) algorithm simulated the working of a prosthesis hand. The subject watched the monitor and sent the motion command by pressing the corresponding keys. Next, he sent new motion command when he judged that the controller has learned the previous command motion completely. Therefore he watched the monitor and also re-sent a teacher signal for the past motion if it was not performed correctly. Figure 7 shows the scheme of this process. After training the system for different trials of each movement, the accuracy of ANFIS based system was evaluated.

Table 3 depicts the results of implemented EMG pattern recognition system using six hand movements previously noted. It presents the acquired results for each subject and the average accuracy of the system for different hand movements. The training process for this system utilized different time durations (response delays) for each individual movement. It varied from three minutes for the hand closing to eleven minutes for the thumb movements.

**Table 3:** Statistical overview of rate of success of the real-time neuro-fuzzy system for sEMG pattern discrimination system

**Movements**							
**Participated subjects**	**Opening**	**Closing**	**Wrist flexion**	**Wrist extension**	**Pinch**	**Thumb flexion**	**Average Results for 6 movements**

**Subject_1**	98%	100%	94%	94%	96%	92%	95.67%
**Subject_2**	98%	100%	100%	96%	98%	94%	97.67%
**Subject_3**	96%	100%	88%	98%	98%	92%	95.33%
**Subject_4**	100%	100%	94%	96%	100%	98%	98%
**Average results**	98%	100%	94%	96%	98%	94%	96.67%
**Standard deviation (STD) for each movements**	1.41%	0%	4.2%	4.2%	1.41%	2.45%	1.18%

The results indicated that the ANFIS system exhibits a superior performance as compared to ANN noted in previous studies [1]. Also the real-time scheme for learning sEMG pattern recognition system with the user evaluation inclusion provided better results. Hence by using the ANFIS real-time learning method, we were able to successfully discriminate among six descriptive and distinct hand movements. To compare the proposed ANFIS method and the simple feed forward ANN approach, the acquired results are presented in Tables 3 and 4, respectively. Based on these results, the average recognition rate for ANFIS and ANN are 96.7% (STD 1.2) and 87.3% (STD 2.6), respectively. These results depict a marked improvement in recognition rate associated to the proposed ANFIS algorithm.

**Table 4:** Statistical overview of rate of success of the real-time ANN system for sEMG pattern discrimination system

**Movements**							
**Participated subjects**	**Opening**	**Closing**	**Wrist flexion**	**Wrist extension**	**Pinch**	**Thumb flexion**	**Average Results for 6 movements**

**Subject_1**	92%	98%	90%	92%	88%	82%	90.33%
**Subject_2**	86%	90%	84%	86%	82%	84%	85.33%
**Subject_3**	90%	94%	88%	86%	80%	78%	86%
**Subject_4**	94%	94%	86%	88%	82%	80%	87.33%
**Average results**	90.5%	94.5%	87%	88%	83%	81%	87.33%
**Standard deviation (STD) for each movements**	2.96%	2.83%	2.24%	2.45%	3.00%	2.24%	2.62%

Confusion matrix, recognized as one of the most useful methods in the study of pattern recognition applications, was utilized for discriminating among various hand movements for all subjects. One benefit of a confusion matrix is that it is easy to verify if the system is confusing two classes (i.e. commonly mislabelling one as another). Table 5 depicts our results for this study. Each column of the matrix represents the instances in an actual class, while each row represents the instances in a predicted class. Results show a minimum class based recognition rate of 94% while a maximum error rate of 5% depicting class confusion was recorded.

**Table 5:** Confusion matrix for identified hand movements for all subject by using ANFIS

**Identified movements Real movements**	**Opening**	**Closing**	**Wrist flexion**	**Wrist extension**	**Pinch**	**Thumb flexion**
**Opening**	**98%**	2%	-	-	-	-
**Closing**	-	**100%**	-	-	-	-
**Wrist flexion**	-	2%	**94%**	4%	-	-
**Wrist extension**	-	-	4%	**96%**	-	-
**Pinch**	-	2%	-	-	**98%**	-
**Thumb flexion**	-	2%	-	-	4%	**94%**

Table 6 depicts two offline and one online based studies for comparison with our findings. As number of hand movements differs in these studies, we defined a criterion which is based on the error percentage per number of movements. Based on the results presented in this table, the best per movement error percentage belongs to our proposed ANFIS algorithm as referenced to our accumulated results in Table 3.

**Table 6:** Comparing the acquired results in this work with previous offline and online EMG pattern recognition system

**Selected Study**	**Number of Hand Movements Used**	**Percentage of Error Range**	**System type**	**Percentage of Error per Movement**
**B. Hudgins**	4	2–30%	Off-line	0.5 – 7.5%
**K. Englehart**	4	6–13%	Off-line	1.5 – 3.25%
**Nishikawa**	10	5.6–9.3%	On-line	0.56 – 0.93%
**This work**	6	2 – 4.67%	On-line	0.33 – 0.78%

Furthermore to evaluate the performance of the proposed ANFIS algorithm, we determined the specificity and sensitivity of each class by using the results depicted in the confusion matrix (Table 5), where the sensitivity and specificity may be defined as number of correct classification of multi-labeled movements per total number of confused cross-class movements, and number of correct classification per number of total hand movements, respectively [24]. Table 7 presents the results for both parameters.

**Table 7:** The statistical characteristics of designed neuro-fuzzy system for recognizing hand movements

**Classifier**	**Specificity**	**Sensitivity**
**ANFIS**	96.67%	97.14%
**ANN**	87.33%	87.07%

## Discussions

sEMG signals provide an extremely useful non-invasive measure of ongoing muscle activity. They are potentially suitable as reference signals for prosthetic hands. Most commercially available hand prostheses have limited range of hand motions. The most natural way to control prosthesis hand would be through a neural based control scheme mediated by the nerves intended for the amputated hand or arm. In this work, we proposed a novel real-time method suitable for hand prosthesis control.

In this non-invasive system, two channel surface mounted electrodes were utilized. The system presented in this work was based on a new intelligent approach, namely a neuro-fuzzy classifier. To train this system, a hybrid method approach consisting of BP and LMS was introduced. Furthermore we employed a subtractive clustering scheme to specify fuzzy system rules.

Also in order to increase recognition rate of this system, two types of features which were time domain and time-frequency representations were used. In time domain we used three major features of sEMG signal namely, MAV, SSC and AR model coefficients and in time-frequency domain, ZC and local maxima of wavelet transform were employed. Once a feature set was constructed, they were fed to a classifier for discriminating amongst six motion commands of selected hand motions. In this study by using a real time learning method, we considered the effect of user evaluation. The training process of the system was continued until its recognition accuracy based on the RMS criterion was satisfied.

After implementing the sEMG pattern recognition system, we acquired classification rate of the proposed system. Our results demonstrated that the utilized real-time based ANFIS approach was superior to the previously introduced ANN scheme as it allowed for a versatile multi motion recognition machine.

## Conclusion

In this work, we introduced a new approach for recognizing sEMG pattern based on real-time neuro-fuzzy system with high degree of correctness. Two types of sEMG features were used, namely time and time-frequency features. Also in order to consider the effect of real-time learning on recognizing six distinct hand movements, an operator based vision feedback approach was utilized. This study demonstrated that ANFIS real-time based learning method is a viable contender in the sEMG pattern recognition system in extending the acceptable range of hand motions intended for hand prosthesis systems.

## Abbreviations

EMG Electromyogram Signal

sEMG Surface Electromyogram Signal

ANFIS Adaptive Neuro-Fuzzy Inference System

BP Back Propagation

LMS Least Mean Square

ANN Artificial Neural Network

AR Autoregressive

MAV Mean Absolute Value

SSC Slope Sign Changes

ZC Zero Crossing

PCA Principle Component Analysis

CMRR Common Mode Rejection Ratio

MF Membership Function

TD Time Domain (features)

TFR Time-Frequency Representations

FIS Fuzzy Inference System

DWT Discrete Wavelet Transform

RMS Root Mean Square

PC Personal Computer

C.G Computer Graphics

## Competing interests

The author(s) declare that they have no competing interests.

## Authors' contributions

MK conceived, designed and implemented the computer model, and drafted the manuscript. MJ supervised the project, contributed to the discussion and interpretation of the results, and participated in manuscript revisions. All authors read and approved the final manuscript.
